# Intrinsically disordered sequences enable modulation of protein phase separation through distributed tyrosine motifs

**DOI:** 10.1074/jbc.M117.800466

**Published:** 2017-09-18

**Authors:** Yuan Lin, Simon L. Currie, Michael K. Rosen

**Affiliations:** From the ‡ Department of Biophysics, University of Texas Southwestern Medical Center and; the § Howard Hughes Medical Institute, Dallas, Texas 75390

**Keywords:** cooperativity, fused in sarcoma (FUS), intrinsically disordered protein, posttranslational modification (PTM), Src homology 3 domain (SH3 domain), RNA granules, liquid–liquid phase separation, multivalency

## Abstract

Liquid–liquid phase separation (LLPS) is thought to contribute to the establishment of many biomolecular condensates, eukaryotic cell structures that concentrate diverse macromolecules but lack a bounding membrane. RNA granules control RNA metabolism and comprise a large class of condensates that are enriched in RNA-binding proteins and RNA molecules. Many RNA granule proteins are composed of both modular domains and intrinsically disordered regions (IDRs) having low amino acid sequence complexity. Phase separation of these molecules likely plays an important role in the generation and stability of RNA granules. To understand how folded domains and IDRs can cooperate to modulate LLPS, we generated a series of engineered proteins. These were based on fusions of an IDR derived from the RNA granule protein FUS (fused in sarcoma) to a multivalent poly-Src homology 3 (SH3) domain protein that phase-separates when mixed with a poly-proline–rich-motif (polyPRM) ligand. We found that the wild-type IDR promotes LLPS of the polySH3–polyPRM system, decreasing the phase separation threshold concentration by 8-fold. Systematic mutation of tyrosine residues in Gly/Ser-Tyr-Gly/Ser motifs of the IDR reduced this effect, depending on the number but not on the position of these substitutions. Mutating all tyrosines to non-aromatic residues or phosphorylating the IDR raised the phase separation threshold above that of the unmodified polySH3–polyPRM pair. These results show that low-complexity IDRs can modulate LLPS both positively and negatively, depending on the degree of aromaticity and phosphorylation status. Our findings provide plausible mechanisms by which these sequences could alter RNA granule properties on evolutionary and cellular timescales.

## Introduction

Eukaryotic cells organize complex biochemical reactions through compartmentalization. In addition to canonical membrane-bound organelles, such as mitochondria and the endoplasmic reticulum, there are also many subcellular compartments that are enriched in selected proteins and nucleic acids but are not surrounded by a lipid bilayer (1). These structures have recently been termed biomolecular condensates to emphasize their common property of concentrating biological molecules (2). Many condensates behave as viscous liquid phases distinct from cytoplasm and nucleoplasm (3–9). A variety of evidence suggests that some may form via the physical process of liquid–liquid phase separation (LLPS)^2^ (2, 9–12). The LLPS model is also supported in part by numerous observations that purified protein and RNA components of condensates can recapitulate the immiscible liquid structures *in vitro* (13–24).

RNA granules, including processing bodies, stress granules, germ line P granules, nuclear speckles, and nucleoli, are a class of condensates that are enriched in RNAs and RNA-binding proteins (25–28). These structures have diverse roles in the metabolism of RNA, including splicing, modification, assembly, storage, degradation, and localization (25–28). Many RNA granule proteins contain multiple RNA-binding domains as well as large intrinsically disordered regions (IDRs) (13, 29–31). The IDRs, as well as the folded domains, are important for assembly of RNA granules (32–36), and their phosphorylation can trigger granule disassembly (37–40). *In vitro*, tandem arrays of folded domains, including RNA-binding domains, can oligomerize and phase-separate when mixed with their multivalent cognate ligands, including repetitive RNAs (13, 14, 24, 41). IDRs can also self-associate and do so through a variety of side-chain interaction modes, including electrostatic, polar, and hydrophobic (42), as well as backbone modes that generate amyloid fibers and potentially other glassy structures (43, 44). These interactions can promote LLPS and/or formation of solid, amyloid-containing hydrogels (12, 16–18, 20–22, 29, 45).

The IDRs of RNA-binding proteins often have low amino acid sequence complexity, being enriched in only a subset of residue types such as Gly, Ser, Tyr, and Gln (29–31). Aromatic residues appear to play particularly important roles in IDR interactions and LLPS. Cation-π interactions between arginine and phenylalanine side chains are thought to be an important driving force for LLPS of the RNA helicase Ddx4 (16). Mutation or deletion of tyrosine residues in the BugZ and Nephrin intracellular domain proteins decreases their propensity to phase-separate (23, 46). Similarly, mutation of tyrosines blocks the formation of hydrogels by the IDR of the RNA-binding protein FUS (29). Tyrosine mutations also block recruitment of IDRs into hydrogels and/or phase-separated liquids formed by the RNA-binding protein hnRNPA2 (47) and FUS (29), as well as into RNA granules in cells (29). The enriched serine residues also likely play important roles in enabling the regulation of IDR interactions, as phosphorylation of the C-terminal domain IDR from RNA polymerase II prevents its recruitment into hydrogels of several IDRs (48).

IDRs and tandem arrays of folded domains can act cooperatively in promoting LLPS when occurring together in the same molecule (17, 20, 21). Such cooperativity is likely important in the formation and regulation of RNA granules, where IDRs as well as folded domains that mediate RNA binding and oligomerization act together with RNAs to produce and maintain the compartments (49, 50). However, the mechanisms underlying IDR-folded domain cooperativity and their potential regulation have not been examined in detail.

In this study, we used the IDR (also called the low-complexity region, residues 1–214) of FUS (referred to simply as FUS hereafter) as a model to investigate how disordered sequences can modulate LLPS of multivalent interacting proteins. The FUS IDR can undergo LLPS on its own at high concentrations (45), and, when fused to the RNA-binding protein PTB, can promote RNA-induced LLPS (17). We found that, when tethered to a polySH3 domain protein that phase-separates when mixed with a cognate poly-proline–rich motif (polyPRM) ligand, wild-type FUS decreases the threshold concentration for LLPS by 8-fold. The magnitude of this effect depends on the number of tyrosine residues in FUS but not on their positions within the sequence. Aromatic residues seem to be particularly important to the effect, as IDR mutants with all tyrosines mutated to leucine or serine do not phase-separate. Conversely, these IDR mutants actively oppose phase separation, increasing the LLPS threshold concentration above that for polySH3 + polyPRM alone. The IDR mutants do not strongly interfere with interactions between polySH3 and polyPRM but, rather, may act by destabilizing the highly concentrated droplet state through entropic effects. Phosphorylated FUS (pFUS) acts similarly to interfere with LLPS of the polySH3–polyPRM system. Our data suggest that the specific amino acid composition of certain IDRs endows them with dual properties of either promoting or opposing LLPS by altering weak self-association of modular domain proteins. These effects could be used in both evolutionary and cellular processes to modulate the existence and properties of RNA granules.

## Results

### Wild-type FUS promotes LLPS of multivalent interacting proteins in cis

**Figure 1.**
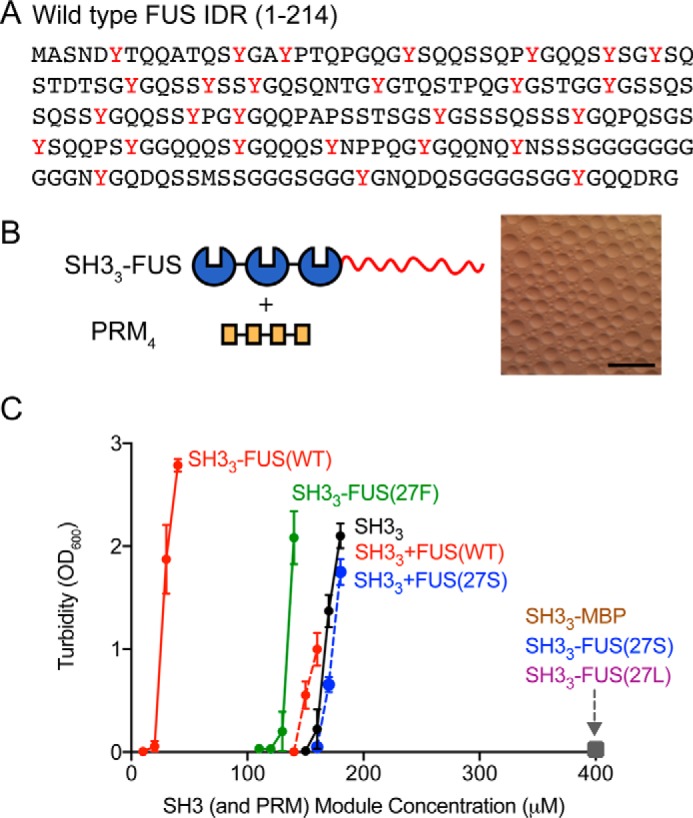
**FUS proteins can promote or oppose LLPS of multivalent interacting proteins.** *A*, primary sequence of wild-type FUS IDR (1–214). The 27 tyrosine residues are shown in *red. B*, liquid droplets observed by bright-field microscopy when 8.3 μm SH3_3_–FUS(WT) and 6.3 μm PRM_4_ were mixed (molecule concentrations). Schematics illustrate the domain structure of the engineered proteins. *Scale bar* = 50 μm. *C*, the effect of the FUS IDR on LLPS of SH3_3_ plus PRM_4_, evaluated by turbidity. *A*_600_ values at the indicated module concentrations and 22 °C are plotted as mean ± S.D., from three independent measurements. The module concentration of SH3 was equal to that of PRM in each sample. SH3_3_ + PRM_4_, *black*; SH3_3_–FUS(WT) + PRM_4_, *red*; SH3_3_–FUS(27F) + PRM_4_, *green*; SH3_3_–FUS(27S) + PRM_4_, SH3_3_–FUS(27L) + PRM_4_, and SH3_3_–MBP + PRM_4_, *gray square*; SH3_3_ + PRM_4_ + FUS(WT) in *trans* with FUS(WT) at concentrations equal to that of SH3_3_, *red dashed line*; SH3_3_ + PRM_4_ + 1.5 mm FUS(27S) in *trans*, *blue dashed line*.

The SH3 domain and its PRM ligand are common modules found in signaling proteins, often in tandem arrays (51, 52). We showed previously that an engineered protein composed of a tandem triplet of SH3 domains (SH3_3_) undergoes LLPS when mixed with a cognate ligand containing four PRM repeats (PRM_4_) (13). This system provided a basis for us to quantitatively examine the effect of fusion of the FUS IDR (Fig. 1*A*) on LLPS of multivalent interacting proteins. We note that engineered molecules containing multiple SH3 domains and IDRs are conceptually analogous to natural RNA-binding proteins containing multiple RNA-binding domains and IDRs but are more tractable biochemically.

We measured the optical density at 600 nm (*A*_600_) of solutions containing PRM_4_ and various SH3_3_–FUS fusion proteins in ratios where the SH3 and PRM module concentrations were equal (Fig. 1*C*). Sharp increases in *A*_600_ corresponded to the appearance of liquid droplets in solution (Fig. 1*B*), enabling us to use *A*_600_ to determine the threshold concentration for phase separation. SH3_3_ and PRM_4_ phase-separated at 160 μm (Fig. 1*C*; except where explicitly noted, all concentrations are stated in terms of module concentration (the total concentration of SH3 domains or PRM elements) so that the molecular concentration of SH3_3_ protein is one-third that of the module concentration). This threshold was unaffected by addition of FUS in *trans* at a protein concentration equal to that of SH3_3_ (Fig. 1*C*). However, tethering wild-type FUS to SH3_3_ (to give SH3_3_–FUS(WT)) decreased the threshold concentration 8-fold, to 20 μm (Fig. 1*C*), consistent with previous reports of cooperativity between IDRs and modular domain interactions in promoting LLPS (17, 20, 21). We note that, although the FUS IDR can also form amyloid-like fibers (29), at room temperature, the transition from droplets to fibers occurs on a timescales of hours (17), much longer than the 10 min used to make measurements here. Thus, our data most likely report solely on LLPS behavior of the proteins.

### Tyrosine residues across FUS make similar contributions to LLPS

**Figure 2.**
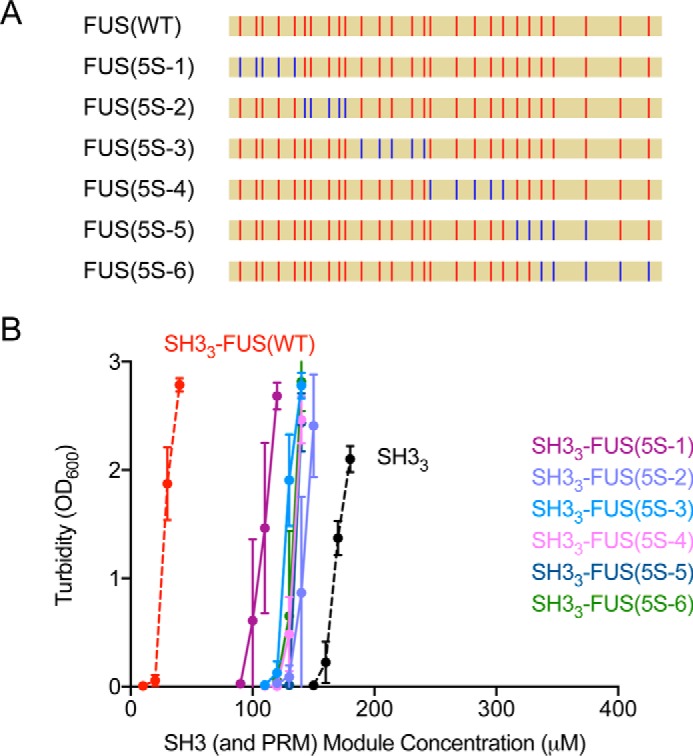
**Tyrosine residues across FUS make similar contributions to LLPS.** *A*, positions of the substitutions of tyrosine by serine in FUS. FUS is shown as *yellow bars*. Tyrosine residues are shown as *red sticks*, and serine residues are shown as *blue sticks. B*, the effect of FUS(5S) mutants on LLPS of SH3_3_ plus PRM_4_, evaluated by turbidity. *A*_600_ at the indicated module concentrations and room temperature (22 °C) are plotted as mean ± S.D. from three independent measurements. The module concentrations of SH3 and PRM are equal in each sample. *Curves* are labeled with legends of the same colors. The *curves* of SH3_3_ plus PRM_4_ and SH3_3_–FUS(WT) plus PRM_4_ are identical to those in Fig. 1*C* and are shown as *black* and *red dashed lines*, respectively, for reference.

The wild-type FUS contains 27 tyrosine residues (Fig. 1*A*). Random mutation of increasing numbers of these tyrosines to serines was shown to progressively decrease recruitment into FUS hydrogels and RNA granules (29). To learn which tyrosine residues in the FUS IDR are responsible for promoting LLPS of the polySH3–polyPRM system, we systematically mutated groups of five consecutive tyrosine residues across the sequence to serine (FUS(5S-1, 2, 3, 4, 5, and 6)) (Fig. 2*A*). We fused these mutants individually to the C terminus of SH3_3_, and used *A*_600_ to determine the phase separation threshold in the presence of PRM_4_. All of the mutants phase-separated at 5- to 6.5-fold higher concentrations than wild-type FUS (Fig. 2*B*). For all but one, LLPS occurred in a narrow window between 120 μm and 130 μm (Fig. 2*B*), indicating that the contribution of tyrosine residues to LLPS is distributed relatively uniformly across FUS. The LLPS threshold for SH3_3_–FUS(5S-1) was 100 μm, slightly lower than that of the others, indicating that the first five tyrosine residues may be somewhat less important than the others (Fig. 2*B*). It is also possible that the adjacent SH3 domains influence the N terminus of FUS and prevent some of the first five tyrosine residues from making interactions that promote LLPS. Nevertheless, these data demonstrate that the aromatic sequence determinants for LLPS are relatively uniformly distributed across the FUS IDR. The number but not the position of aromatic residues determines the LLPS threshold. This behavior mirrors that of LLPS by multimodular domain proteins alone, where the phase separation threshold is dependent on domain valency (13, 15), suggesting conceptual similarity between the two molecular types.

### IDR mutants with all aromatic residues substituted oppose LLPS

To determine what residue types could function analogously to tyrosines in FUS to promote LLPS, we generated a series of mutants in which all 27 tyrosine residues were mutated to phenylalanine, leucine, or serine (FUS(27F), FUS(27L), and FUS(27S)). We fused the mutants to the C terminus of SH3_3_ and determined the LLPS threshold in the presence of PRM_4_ (Fig. 1*C* and supplemental Fig. S1). FUS(27F) promoted LLPS analogously to wild-type FUS, but much less strongly, decreasing the threshold only to 120 μm (*versus* 160 μm for SH3_3_ + PRM_4_ alone). The other two mutants behaved quite differently in two respects. First, neither promoted LLPS, including FUS(27L), which would be classified as more hydrophobic than wild-type FUS in many hydrophobicity scales (53–55). FUS(27F) would also be classified as more hydrophobic than the wild type, but it too is less effective in promoting LLPS. Even in alternative hydrophobicity scales that classify tyrosine as extremely hydrophobic, leucine is placed to be more hydrophobic than phenylalanine (56, 57). Thus, promotion of LLPS does not appear to be dependent on simple hydrophobicity but, rather, specifically on aromaticity.

Secondly, the additional mutants were all unexpectedly inhibitory toward phase separation; in all cases, the solutions remained clear, and no LLPS or precipitation was observed up to 400 μm, the highest concentration examined. Thus, if their aromatic content is lost, then low-complexity sequences can decrease the drive for phase separation by multimodular domain proteins. Notably, FUS(27S) does not alter the phase separation threshold concentration for SH3_3_ + PRM_4_ when added in *trans*, even at concentrations as high as 1.5 mm (Fig. 1*C*). The opposing effect of FUS(27S) must thus be applied in *cis*. Together, these results indicate that the promotion of LLPS by FUS is mediated by specific interactions involving aromatic residues and that the LLPS-opposing effect is general to non-aromatic substitutions, both polar and hydrophobic.

### FUS(27S) does not strongly interfere with interactions between SH3 and PRM

**Figure 3.**
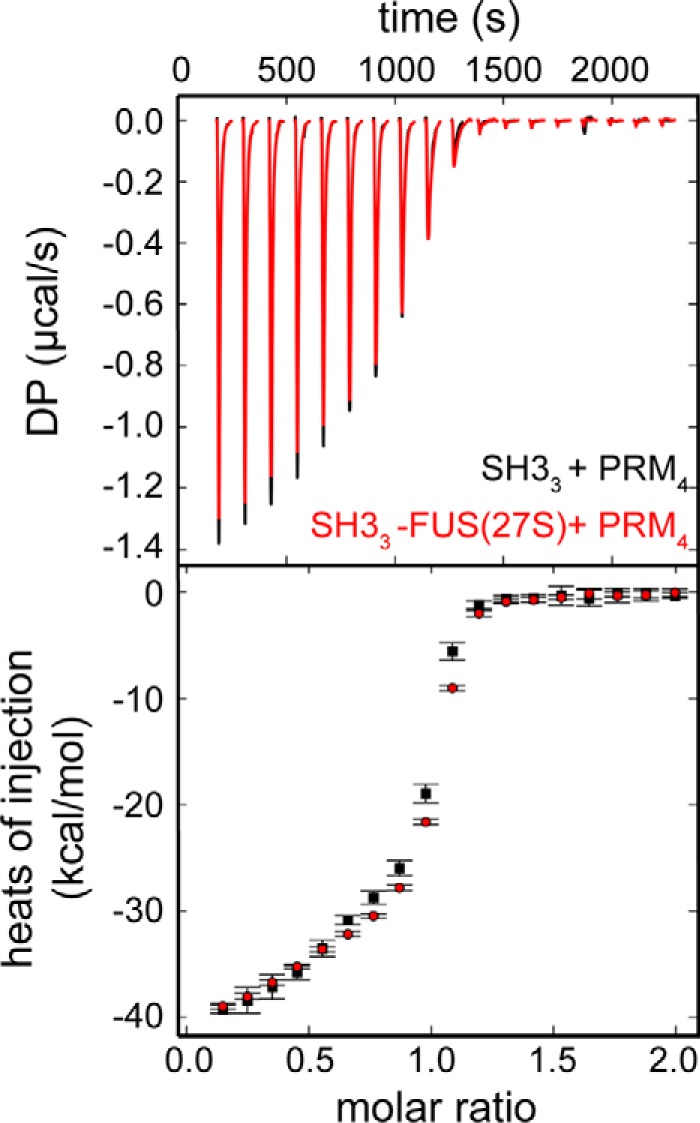
**FUS(27S) does not interfere with the interaction between SH3 and PRM.** Isothermal titration calorimetry analysis of the binding of SH3_3_ or SH3_3_–FUS(27S) to PRM_4_ was performed. ∼200 μm PRM_4_ was titrated into 20 μm SH3_3_ (*black*) or SH3_3_-FUS(27S) (*red*) (molecule concentration). At such concentrations, phase separation did not occur. A thermogram (*top panel*) and isotherm (*bottom panel*) are shown. *DP*, differential power.

Next we sought to understand how FUS(27S) opposes LLPS by the polySH3–polyPRM system. One possibility is that FUS(27S) interferes with the interaction between SH3 and PRM that normally allows the proteins to oligomerize. To test this hypothesis, we used isothermal titration calorimetry to examine binding of SH3_3_ to PRM_4_ with or without the attachment of FUS(27S) or FUS(27L) to SH3_3_. Given the multivalent nature of the engineered proteins and the resulting complicated patterns of association, the isotherms could not be fit with a simple model. Nevertheless, the thermograms and isotherms under all three conditions were nearly identical, demonstrating that the interaction between SH3 and PRM was not strongly affected by FUS(27S) or FUS(27L) (Fig. 3 and supplemental Fig. S2).

### FUS(27S) alters the self-association properties of the tethered proteins

In addition to oligomerization, LLPS of multivalent molecules is also modulated by the intrinsic solubilities of the interacting species; that is, their propensities to interact weakly with themselves over solvent (58, 59). Such weak self–self interactions are often assessed through the scattering second virial coefficient (A_2_), which is closely related to the osmotic second virial coefficient (B_22_) under dilute buffer conditions (60). These parameters reflect the deviation from ideal solution behavior because of pairwise interactions between solute molecules in a solution (61). For a variety of proteins, A_2_ correlates reasonably well with solubility, defined as the concentration of protein in a bulk solution phase that is in equilibrium with protein crystals or a condensed protein liquid phase (62–66). In the latter case, this corresponds to the threshold concentration for LLPS. Further, the “diffusion interaction parameter,” k_D_, the linear change in protein diffusion coefficient with concentration, is a major component of B_22_ (67, 68). Negative values of k_D_ (negatively correlated diffusion coefficient and concentration) correspond to favorable relative self–self interactions, and more negative values have been shown to correlate with increased propensity for LLPS (69–74).

**Figure 4.**
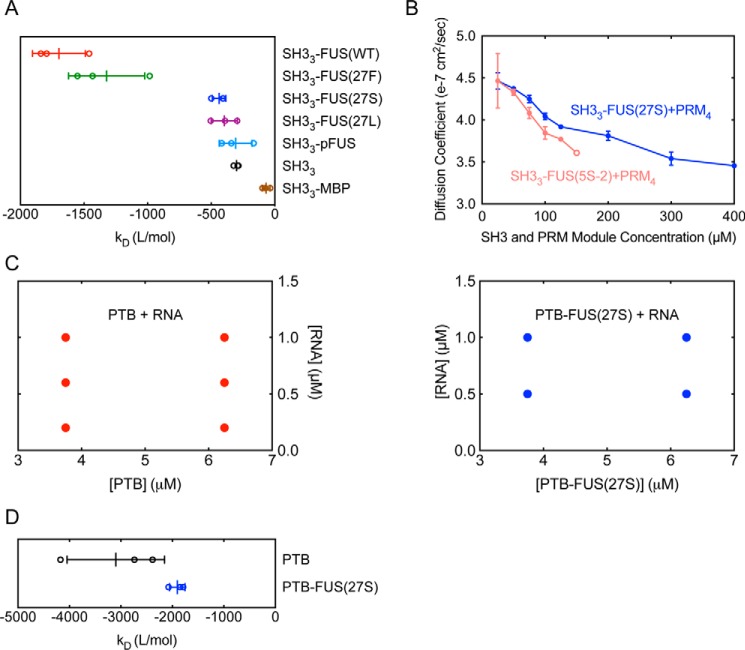
**FUS mutants oppose phase separation by altering self-associations of the entire molecules.** *A*, k_D_ values of SH3_3_–FUS(WT) (*red*), SH3_3_–FUS(27F) (*green*), SH3_3_–FUS(27S) (*blue*), SH3_3_–FUS(27L) (*purple*), SH3_3_–pFUS (*light blue*), SH3_3_ (*black*), and SH3_3_–MBP (*brown*) obtained from DLS experiments (means of three independent replicates ± S.D.). *B*, diffusion coefficients of SH3_3_–FUS(27S) + PRM_4_ (*blue circles*) and SH3_3_–FUS(5S-2) + PRM_4_ (*light red circles*) at different equal module concentrations, measured by DLS (means of three independent replicates, ± S.D.). The *light red open circle* indicates the occurrence of phase separation, and the diffusion coefficient was determined for the supernatant, which was separated from the droplets by centrifugation at 15,000 × *g* for 5 min. *C*, FUS(27S) opposes LLPS of PTB + RNA. PTB or PTB–FUS(27S) was mixed with RNA at the indicated molecule concentrations and examined with microscopy. *Red dots* indicate the presence of liquid droplets, and *blue dots* indicate no LLPS. *D*, k_D_ values of PTB (*black*) and PTB-FUS(27S) (*blue*) measured by DLS (means of three independent replicates, ± S.D.).

The requirement of FUS(27S) being in *cis* to oppose LLPS suggests that FUS(27S) might act through altering the self-association properties of SH3_3_–FUS. To test this hypothesis, we used dynamic light scattering (DLS) and static light scattering (SLS) to measure the diffusion coefficients and scattering properties of SH3_3_–FUS proteins as a function of concentration. The relationship between diffusion coefficient (D) and protein concentration (c) can be approximately described by *D* = *D*_0_ (1+*k_D_c*), where D_0_ is the diffusion coefficient at infinite dilution. We obtained k_D_ values by fitting the concentration-dependent diffusion coefficients (supplemental Fig. S3) to this equation. The k_D_ values of SH3_3_–FUS(WT) and SH3_3_–FUS(27F) were more negative than that of SH3_3_, indicating stronger self-interaction (Fig. 4*A*). In contrast, the k_D_ of SH3_3_–FUS(27S) and SH3_3_–FUS(27L) were both less negative, indicating weaker self-interaction (Fig. 4*A*). A_2_ values were similarly calculated from the concentration dependence of scattering intensities, and the A_2_ values of the four proteins showed an analogous pattern (supplemental Fig. S5*A*). The difference of k_D_ and A_2_ between the different fusion proteins correlates with their phase separation behaviors: FUS proteins that have stronger self-association undergo LLPS with PRM_4_ at a lower threshold concentration, whereas those that have weaker self-association produce a higher threshold concentration. DLS experiments also showed that, as concentration increased, although mixtures of SH3_3_–FUS(27S) and PRM_4_ did not undergo LLPS, they could still form larger complexes (inferred from smaller D values) than those required for phase separation of SH3_3_–FUS(5S-2) + PRM_4_ (Fig. 4*B*). With the caveat that D can reflect properties other than mass, these data are consistent with the idea that weaker self-association (and, by inference, higher solubility) opposes phase separation, even though oligomerization through SH3–PRM interactions is largely unaffected.

To test whether the effect of FUS(27S) could be general to other proteins, we fused the protein to the C terminus of PTB, an RNA-binding protein bearing four RNA recognition motifs (termed PTB-FUS(27S)). We showed previously that PTB undergoes LLPS when mixed with RNA and that fusion of PTB to FUS(WT) promotes this behavior (13, 17). In contrast, attachment of FUS(27S) opposed LLPS of PTB + RNA (Fig. 4*C*). DLS and SLS experiments revealed that both k_D_ and A_2_ values of PTB-FUS(27S) were less negative than those of PTB alone, suggesting that PTB-FUS(27S) exhibited weaker self-association (Fig. 4*D* and supplemental Figs. S4 and S5*B*). Together, our data suggest that FUS(27S) is not a highly specific LLPS inhibitor but may be able to generally reduce the self-association of tethered proteins and thus oppose their LLPS.

This conclusion raises the possibility that such opposing effects might not be specific to FUS mutants but could be exhibited by other elements that similarly alter the self-association property of the entire conjugated molecules. To test this possibility, we fused the maltose-binding protein (MBP) to the C terminus of SH3_3_ (SH3_3_–MBP). MBP is well-known to improve the biochemical behaviors of proteins it is fused to, often enabling low-solubility proteins to remain in solution at higher concentrations (75). Indeed, DLS experiments indicated that k_D_ of SH3_3_–MBP was much less negative than that of other SH3_3_ fusion proteins, suggesting very weak self-association (Fig. 4*A*). Correspondingly, SH3_3_–MBP did not undergo LLPS with PRM_4_ at 400 μm concentration (Fig. 1*C*), demonstrating that MBP, like the FUS mutants, was sufficient to oppose LLPS of SH3_3_ + PRM_4_.

### Phosphorylation of FUS disassembles liquid droplets

**Figure 5.**
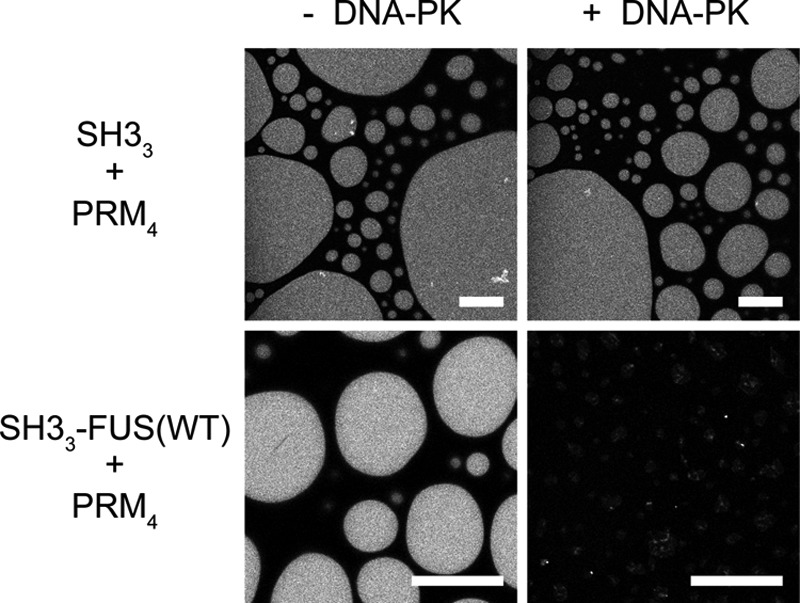
**Phosphorylation of FUS opposes LLPS.** Fluorescence microscopy images of droplets formed by 83.3 μm SH3_3_ + 62.5 μm PRM_4_ and 16.7 μm SH3_3_-FUS(WT)+12.5 μm PRM_4_ (molecule concentration) in the presence or absence of DNA-PK. SH3_3_ and SH3_3_–FUS(WT) were labeled with Oregon Green 488. Fluorescence images were taken 3 h after initiating the reaction. Images were acquired with identical microscope settings and are shown with the same brightness and contrast. *Scale bars* = 20 μm.

FUS was identified previously as the substrate of DNA-dependent protein kinase (DNA-PK) *in vitro* (76). The four serine residues in the FUS IDR reported to be phosphorylated by DNA-PK (Ser-26, Ser-42, Ser-61, and Ser-84) are in close proximity to tyrosine residues (Tyr-25, Tyr-41, Tyr-58, and Tyr-81, respectively) (77), and phosphorylation impedes the formation of FUS hydrogels and targeting of FUS to preformed hydrogels (77). To test whether phosphorylation of FUS can affect LLPS of SH3_3_ + PRM_4_, we incubated liquid droplets formed by SH3_3_–FUS(WT) plus PRM_4_ with recombinant DNA-PK, Mg^2+^, and ATP for 3 h. The liquid droplets disassembled almost completely by the end of the incubation period, whereas liquid droplets formed by SH3_3_ (without FUS attachment) plus PRM_4_ were not affected by DNA-PK (Fig. 5). We further demonstrated that only SH3_3_–FUS(WT) but not SH3_3_ can be highly phosphorylated by DNA-PK, confirming that the effects of the kinase are applied through the FUS IDR and specific to the FUS-containing system (supplemental Fig. S6). DLS experiments further showed that the k_D_ of SH3_3_–FUS(WT) became less negative upon phosphorylation (creating SH3_3_–pFUS) (Fig. 4*A*). This result suggests that phosphorylation of FUS can disassemble liquid droplets by weakening the self-association of SH3_3_–FUS(WT). Thus, mutation of tyrosine residues and phosphorylation of proximal serine residues appear to act through similar mechanisms to disfavor LLPS.

## Discussion

Previous data suggested that LLPS contributes to the formation of RNA granules through cooperativity between multivalent RNA-binding domains, RNAs, and IDRs (17, 20–22). Our data here suggest that the ability of IDRs to promote LLPS may be general for multivalent proteins, as wild-type FUS, when tethered to SH3_3_, can decrease the LLPS threshold concentration in the presence of PRM_4_ ∼8 fold. Because the FUS IDR contains only five acidic residues and one basic residue (Fig. 1*A*), it is unlikely that charge effects primarily drive this behavior. Rather, as in the ability of FUS to form hydrogels and be retained by hydrogels (29), and as observed for LLPS by other IDRs (16, 23, 46), the numerous tyrosine residues in FUS are important for LLPS. Our systematic investigation of these tyrosine residues reveals that they are broadly involved in and contribute nearly equally to LLPS (at least at the resolution of five tyrosines). These observations are consistent with a previous NMR study on FUS indicating that the backbones of all residues display slower motions upon LLPS, suggesting distributed interactions across the length of the polypeptide chain (45). In contrast to this behavior of FUS, the IDR of hnRNPA2 binds to hydrogels and partitions into liquid droplets through Tyr/Phe interactions that are less distributed, with some positions contributing substantially more than others (47). It remains to be seen whether FUS-like (distributed) or hnRNPA2-like (focused) aromatic interactions are more prevalent in promoting LLPS and related behaviors of other IDRs.

Analyses of both natural IDRs, such as elastin (78), and engineered IDRs composed of repeated sequence motifs (79) have suggested that sequence hydrophobicity plays an important role in determining the driving force for LLPS. For FUS, however, hydrophobicity does not appear to be sufficient to enhance LLPS of polySH3–polyPRM. Although there is no single best method for assessing amino acid hydrophobicity, all scales classify Tyr, Phe, and Leu as appreciably hydrophobic (53, 54, 80). Yet clearly Tyr and Phe act differently than Leu in modulating LLPS. The difference between Tyr/Phe and Leu suggests the likely importance of interactions such as π–π, CH–π, OH–π, and NH–π, provided by aromatic side chains, in promoting LLPS (81). Although the difference in interaction energy between a Phe pair and a Tyr pair can be minor in certain context (82), other studies have shown that the OH group in the phenol ring of Tyr enhances π-π and CH-π interactions (83, 84). This could potentially explain why Tyr is better than Phe at promoting LLPS. Additionally, the ability of the residues to organize bound water molecules is likely to play an important role, as loss of such waters is a major driving force for LLPS of elastin (85). Further biophysical studies are required to understand the importance of aromatic interactions in FUS.

In the series of FUS mutants, the effects on LLPS parallel the k_D_ and A_2_ properties of the SH3_3_–FUS fusions. The proteins that promote LLPS more strongly have more negative k_D_ values and A_2_ values of less than zero (Fig. 4*A* and supplemental Fig. S3), both of which indicate stronger self-interaction. Thus, it is likely that the differential self-interaction of the FUS fusions confers differential effects on LLPS in the presence of PRM_4_.

It is less clear, however, why the FUS(27L) and FUS(27S) as fusion partners are not neutral toward LLPS of polySH3–polyPRM but are actively inhibitory. A variety of studies have examined the ability of fusion partners, including MBP (75) and highly soluble IDRs (86, 87), to increase the soluble expression and decrease the aggregation of proteins (which, for ease of description we will call “hosts” here). In related work, numerous experimental and computational studies have examined LLPS of elastin-like peptides (ELPs), which are sequence variants of natural elastin that phase-separate in response to changes in temperature and solvent conditions (85, 88). Many studies have examined the effects of joining folded domains or IDRs as fusion partners to ELP hosts (89–91). The analogy to our system is not exact because LLPS of ELPs is favored by increasing temperatures, in contrast to our system, where LLPS is favored by lower temperatures (supplemental Fig. S7). Nevertheless, as in our system, it has been observed that some fusion partners inhibit phase separation relative to the unfused ELP hosts.

The mechanisms by which this inhibition of self-association/LLPS occurs remain unclear. Several non-mutually exclusive possibilities have been proposed. First, electrostatic repulsion by highly charged fusion partners may keep the host chains apart, preventing aggregation (91, 92). Second, fusion partners have been proposed to bind aggregation-prone sites, often partially unfolded regions that exist during folding, of hosts to prevent self-association (75). Third, in ELPs, the release of organized water from hydrophobic surfaces is known to be a principal driver of LLPS, and incorporation of charged residues in the ELP chain destabilizes those immobilized waters, decreasing the magnitude of this entropic effect (85, 93). It has been proposed that charged fusion partners could act similarly to decrease the driving force for LLPS of ELP hosts (91). Fourth, in a so-called entropic bristle effect, fusion partners occupy a large space that is restricted when other proteins bind the host, thus decreasing host self-association (86, 94). Although this effect is most often cited for IDR fusion partners, it could also occur with folded partners. Finally, ELP chains collapse upon LLPS, which produces an unfavorable decrease in configurational entropy. It has been proposed based on molecular dynamics simulations that non-interacting chains fused to the terminus of an ELP host would also lose configurational entropy themselves upon LLPS, increasing the size of this energetic penalty (95).

These same effects could, in principle, affect LLPS of the SH3–FUS–PRM system here, which depends on weak interactions between soluble oligomers. Of these mechanisms, the first seems unlikely because FUS has little charge. The second is also unlikely, as the inhibitory effects are seen for both SH3_3_ and PTB hosts, and, moreover, both systems involve well-folded domains that are unlikely to have substantial unfolded, aggregation-prone elements. The third also seems unlikely because release of water probably plays a lesser role in self-association/LLPS of highly hydrophilic folded domains than in highly hydrophobic ELPs. Moreover, it is not clear that fusing a chain to one end of even a hydrophobic polymer would have the same influence on bound waters as interspersing charged residues into the polymer. Thus, the fourth and fifth mechanistic possibilities, both involving nonspecific entropic effects, the former applicable to folded domain and IDR fusion partners, and the latter only to IDRs that collapse upon LLPS, seem most likely in our system.

In this view, fusion of an IDR to a host protein could have two limiting consequences. Nonspecific entropic effects will inherently disfavor LLPS. Oppositely, interactions between fusion partners will favor LLPS. The net effect of any given fusion partner will then be the balance of these two opposing forces. For FUS(27S) and FUS(27L) and phosphorylated FUS (pFUS), the former effect dominates (in pFUS, with potentially repulsive electrostatic effect because of phosphate groups, as in mechanism 1 above), but for FUS(WT) and FUS(27F), aromatic motifs provide sufficient adhesion to overcome unfavorable entropic effects, and the net effect is favorable.

In the polySH3 system, the entropic bristle effect likely manifests only at high protein concentrations because the k_D_ and A_2_ values do not differ between the FUS(27S) or FUS(27L) fusion proteins and the unfused polySH3 host, at least at the relatively low concentration range we tested (Fig. 4*A* and supplemental Fig. S5*A*). Further, fusion to FUS(27S) or FUS(27L) does not alter high-affinity binding between polySH3 and polyPRM (Fig. 3 and supplemental Fig. S2). But the entropic bristle effect could destabilize the droplet state (326 μm SH3_3_ within droplets (supplemental Fig. S8) *versus* 7 μm in the surrounding bulk (Fig. 1*C*)), where excluded volume effects should be larger. For PTB, the effect is also manifest in the dilute state, as shown by the changes in k_D_ and A_2_ values upon fusion to FUS(27S) (Fig. 4*D* and supplemental Fig. S5*B*). There, the entropic bristle effect may involve weakening of PTB–RNA interactions and/or PTB–PTB interactions that govern solubility.

In general, any element that changes the apparent self-association of oligomers has the potential to modulate phase separation and might be exploited by biological systems. First, our mutagenesis experiments reveal the importance of aromatic residues in promoting LLPS. The hydrophilic residues might be critical in opposing LLPS. Evolution could modulate the propensity of a molecule to undergo LLPS by changing the number of these residues as well as other residues in various contexts. A recent study has demonstrated that the usage of aliphatic residues in the low complexity region of Pab1, an RNA-binding protein in stress granules, and thus the hydrophobicity of this region, are shaped by natural selection (96). We notice that, in this study, similar to our observation, substitution of these aliphatic residues can either promote or repress LLPS of Pab1. Second, we have shown that phosphorylation of FUS can have a similar effect as direct substitution of aromatic residues (Fig. 5). Other posttranslational modifications of IDRs could conceivably do the same, and in other systems, phosphorylation could increase self-association, enhancing LLPS, as observed in SH2 domain-based signaling pathways (15, 41, 97). Further, in contrast to the covalently tethered MBP in our synthetic construct, a naturally occurring protein could non-covalently bind to phase-separated proteins in cells and promote or oppose LLPS. In fact, the latter has been observed in two systems that we are aware of. In the engineered fusion of an ELP host with the protein tendamistat, binding of the large polar protein porcine pancreatic α-amylase inhibits LLPS (91). In a more physiologically relevant example, the cytomegalovirus protein IE1 disrupts the phase-separated biomolecular condensate PML nuclear bodies (98). IE1 utilizes an N-terminal coiled-coil domain to bind directly to the PML protein, which is the essential scaffold of the PML nuclear bodies. However, the coiled-coil domain is unable to disrupt PML bodies. Rather, disruption requires the 20-kDa C-terminal domain of IE1, which is highly negatively charged and predicted to be highly disordered. Although the mechanism of the disruptive activity of IE1 has not been explored, the framework developed here suggests that the protein could act through using its coiled-coil domain to recruit its destabilizing C-terminal IDP to PML. As more factors are discovered that modulate the dynamics of biomolecular condensates, this physical mechanism may be more widely observed.

Our observation highlights two linked processes involved in LLPS of biomolecules: the polymerization of molecules (usually mediated by specific, relatively strong, and multivalent interactions) and the demixing of the resulting oligomers out of bulk solution (mediated by weak but numerous solute–solute interactions that compete with solute–solvent interactions). The advantage of IDRs as modulator of LLPS lies in their versatile physical properties, which rely on their special amino acid compositions with three elements: the aromatic residues mediate short-range, aromatic interactions and promote LLPS; the abundant hydrophilic residues dominate the solubility of IDRs and oppose LLPS when aromatic interactions are lost; and the abundant serine and tyrosine residues can be readily phosphorylated, enabling rapid transitions between promoting and opposing LLPS. These properties should enable IDRs to modulate LLPS through evolutionary changes (by addition to or loss from a multidomain partner or by addition or loss of aromatic residues in the sequence) as well as during cellular processes involving alterations in the balance of kinase and phosphatase activities. These mechanisms could play important roles in controlling the physical properties, assembly/disassembly, and functions of cellular structures such as RNA granules.

## Experimental procedures

### DNA constructs and reagents

For the SH3_3_, SH3_3_–FUS, and SH3_3_–MBP constructs, SH3_3_, SH3_3_–FUS, or SH3_3_–MBP was inserted into an engineered pGex vector in which a tobacco etch virus (TEV) protease cleavage site was inserted after a GST tag. The restriction sites were NdeI and BamHI. For the PTB and PTB–FUS(27S) constructs, the vector was an engineered pet11a vector in which a TEV cleavage site was inserted after a His_6_ tag. The restrictions sites were NdeI and BamHI. For the PRM_4_, FUS(WT), and FUS(27S) constructs, the vector was an engineered pMal-c2 vector in which a TEV cleavage site was inserted after MBP and a C-terminal TEV-cleavable His_6_ tag was inserted right before the stop codon. The restrictions sites were NdeI and BamHI. RNA was synthesized by Integrated DNA Technologies, Inc. See also supplemental Table S1 for the protein and RNA sequences.

### Protein expression and purification

All proteins were expressed in *Escherichia coli* strain BL21(DE3)T1^R^ (Sigma-Aldrich). Bacteria were grown in LB at 37 °C and induced at *A*_600_ 0.6–1.0 with 1 mm isopropyl 1-thio-β-d-galactopyranoside at 18 °C for 16 h. Cells were lysed by homogenization (EmulsiFlex-C5, Avestin) and followed by centrifugation at 50,000 × *g* for 30 min at 4 °C.

For SH3_3_, SH3_3_–FUS, and SH3_3_–MBP, the proteins were purified with glutathione-Sepharose resins (New England Biolabs). The GST tag was cleaved by TEV protease. Proteins were further purified with a Source 15Q anion exchange column, followed by a Superdex 200 column equilibrated in a buffer of 150 mm KCl, 10 mm imidazole (pH 7.0), 1 mm EGTA, 1 mm MgCl_2_, and 1 mm DTT. The proteins were flash-frozen in liquid nitrogen and stored at −80 °C. All assays for these proteins were performed in the same buffer, except where otherwise indicated.

The PRM_4_ protein was purified with Ni-NTA resins (Qiagen). The MBP and His_6_ tags were removed by TEV protease. The protein was then purified with a Source 15S cation exchange column, followed by a Superdex 75 column equilibrated in a buffer of 150 mm KCl, 10 mm imidazole (pH 7.0), 1 mm EGTA, 1 mm MgCl_2_, and 1 mm DTT. The protein was flash-frozen in liquid nitrogen and stored at −80 °C.

FUS(WT) and FUS(27S) were first purified with Ni-NTA resin (Qiagen), followed by amylose affinity purification (New England Biolabs). The MBP and His_6_ tags were removed by TEV protease. MBP tags were purified away by incubating the samples with amylose resin. The flow-through was further purified by a Superdex 75 column equilibrated in a buffer of 150 mm KCl, 10 mm imidazole (pH 7.0), 1 mm EGTA, 1 mm MgCl_2_, and 1 mm DTT to remove the His_6_ tag.

For PTB and PTB–FUS(27S), proteins were purified with Ni-NTA resins (Qiagen). 1.5 m NaCl was included in the wash buffer to remove DNA/RNA binding. The His_6_ tag was removed by TEV protease. The proteins were further purified with a Source 15S cation exchange column, followed by a Superdex 200 column equilibrated in a buffer of 150 mm KCl, 10 mm imidazole (pH 7.0), 1 mm EGTA, 1 mm MgCl_2_, and 1 mm DTT. The proteins were flash-frozen in liquid nitrogen and stored at −80 °C. The concentrations of all proteins were measured from the absorbance at 280 nm on a Nanodrop 1000 device, and the extinction coefficients were obtained from ExPASy ProtParam.

### Turbidity assay

SH3_3_, SH3_3_–FUS, or SH3_3_–MBP was mixed with PRM_4_ at the indicated temperatures and concentrations of SH3 and PRM modules. All samples contained the same SH3 and PRM module concentrations. For the in *trans* experiments, FUS(WT) was added with the same concentration of SH3_3_, and FUS(27S) was added with a constant concentration of 1.5 mm. Ten minutes after mixing, the optical density of the samples at 600 nm was measured with an Agilent 8453 UV-visible spectroscopy device with a 1-cm path length. The turbidity was measured as optical density and plotted as mean ± S.D. from three independent measurements. The lowest concentration at which the mean of optical density is above an arbitrarily chosen value 0.05 is regarded as the threshold concentration of LLPS.

### Isothermal titration calorimetry

Proteins were dialyzed in the same buffer (150 mm KCl, 10 mm imidazole (pH 7.0), 1 mm EGTA, 1 mm MgCl_2_, and 1 mm tris(2-carboxyethyl)phosphine) overnight before isothermal calorimetry measurements. Their concentrations were determined by absorbance at 280 nm with an Agilent 8453 UV-visible spectrometer. Measurements were performed at 20 °C on an iTC200 instrument from GE Healthcare. 20 μm SH3_3_, SH3_3_–FUS(27S), or SH3_3_–FUS(27L) (molecule concentration) was loaded into the cell, and 200 μm PRM_4_ (molecule concentration) was loaded in the syringe and titrated into the cell. Each injection contained 2 μl PRM_4_. The time interval between injections was 120 s so that the system could come to equilibrium after each injection. Isotherms were generated using the NITPIC software.

### Dynamic and static light scattering

Before the measurements, all proteins were filtered through an ultrafree-MC GV centrifugal filter with a 0.22-μm pore size (EMD Millipore) and centrifuged at 16,000 × *g* for 10 min to remove potential aggregations and dust. The experiments were performed at 25 °C on a DynaPro NanoStar instrument (Wyatt Technology). Dynamic and static light scattering of samples at the indicated molecule molar concentrations were measured simultaneously. The diffusion coefficients were measured over 10 acquisitions, each with a 5-s acquisition time, and three technical replicates for each concentration and analyzed using Dynamics software (Wyatt Technology). The diffusion interaction parameter k_D_ was obtained by fitting the diffusion coefficients to *D* = *D*_0_ (1+*k_D_c*). Scattering second virial coefficients, A_2_, were directly reported by Dynamics software, based on the concentration dependence of scattering intensities, and converted to the unit of milliliters per gram by multiplying molecular weight. For each protein, the above procedure was repeated for three independent samples. k_D_ and A_2_ were plotted as mean ± S.D.

### Phase separation assay

PTB or PTB–FUS(27S) was mixed with RNA at the indicated molecule concentrations and at room temperature (22 °C). The occurrence of phase separation was evaluated by visually examining the solution for liquid droplets with a Nikon SMZ1500 microscope.

### Phosphorylation of SH3_3_–FUS(WT)+PRM_4_ by DNA-PK and imaging

A concentration of 16.7 μm SH3_3_–FUS(WT) was mixed with 12.5 μm PRM_4_ (molecule not module concentration) with or without 114.1 units of DNA-PK (Promega, V5811) at room temperature (22 °C). One unit is defined as the amount of enzyme required to incorporate 1 pmol of phosphate into DNA-PK peptide substrate in 1 min at 30 °C. The reaction buffer contained 150 mm KCl, 10 mm imidazole (pH 7.0), 1 mm EGTA, 10 mm MgCl_2_, 1 mm DTT, 400 μm ATP, and 10 ng/μl linearized double-stranded DNA. To visualize the droplets with fluorescence microscopy, 200 nm SH3_3_–FUS(WT) chemically labeled with Oregon Green 488 was included in the samples. As a control, 83.3 μm SH3_3_ was mixed 62.5 μm PRM_4_ with or without 114.1 units DNA-PK. SH3_3_ was labeled with Oregon Green 488. All concentrations refer to molecular but not module concentrations. The samples were placed in a glass-bottom chamber coated with 3% BSA and washed three times with H_2_O. Images were taken 3 h after the initiation of the reaction with a Leica TCS SP8 laser-scanning confocal microscope.

### Phosphorylation of SH3_3_–FUS(WT) by DNA-PK and Western blotting

A concentration of 30 μm SH3_3_ or SH3_3_–FUS(WT) (molecule not module concentration) was mixed with or without 245.6 units of DNA-PK (Promega, V5811) at 30 °C for 30 min. One unit is defined as the amount of enzyme required to incorporate 1 pmol of phosphate into DNA-PK peptide substrate in 1 min at 30 °C. The reaction buffer contained 50 mm KCl, 20 mm HEPES (pH 7.5), 10 mm MgCl_2_, 0.2 mm EGTA, 0.1 mm EDTA, 1 mm DTT, 800 μm ATP, and 10 ng/μl linearized double-stranded DNA. The samples were analyzed either by SDS-PAGE gel followed by Coomassie Blue staining or Western blotting. For Western blotting, phosphoserine/phosphothreonine/phosphotyrosine antibody (SPM101, Novus Biologicals, NB600–558SS, with a 1:50 dilution factor) was used to detect phosphorylation. Secondary antibody was a mouse IgGκ binding protein conjugated to HRP (mIgGκ BP-HRP, sc-516102, Santa Cruz Biotechnology, 1:10,000 dilution factor).

### Determination of droplet concentration of SH3_3_–FUS(WT)

A concentration of 20 μm SH3_3_–FUS(WT), 50 nm SH3_3_–FUS(WT) labeled with Oregon Green 488, and 15 μm PRM_4_ (molecule but not module concentration) was mixed to undergo LLPS. After 1 h incubation, the images of droplets were taken on a Leica-based spinning disk confocal microscope (electron-multiplying charge-coupled device (EMCCD) digital camera, ImageEM X2, Hamamatsu; confocal scanner unit, CSU-X1, Yokogawa). Background intensity was removed by subtracting the image of buffer alone. The image of a homogenous solution containing 2 μm Oregon Green 488 was taken under the same illumination condition. The maximum intensity of this image was used to divide the whole image. The resulting image was used to divide all images for correction of uneven illumination. The intensities at the center of the droplets were counted as the droplet intensities, which were then converted into the absolute concentration of labeled SH3_3_–FUS(WT) within droplets based on a standard curve generated by a series of pure Oregon Green 488 at 0.05, 0.10, 0.25, 0.50, 0.75, 1.00, 2.00, and 4.00 μm. The final droplet concentration of labeled SH3_3_–FUS(WT) was the mean of 30 droplets from three independent samples. Because the ratio between total labeled and total unlabeled SH3_3_–FUS(WT) in the solution was known, the droplet concentration of unlabeled SH3_3_–FUS(WT) can be calculated from that of labeled SH3_3_– FUS(WT).

## Author contributions

Y. L. and M. K. R. conceived the project. Y. L. designed and conducted the experiments. S. L. C. also conducted experiments. Y. L. and M. K. R. analyzed the data and wrote the manuscript, which S. L. C. helped revise.

## Supplementary Material

Supplemental Data

## Footnotes

2 The abbreviations used are:

LLPS: liquid–liquid phase separation
IDR: intrinsically disordered region
FUS: fused in sarcoma
PTB: polypyrimidine tract–binding protein
SH3: Src homology 3
PRM: proline-rich motif
DLS: dynamic light scattering
SLS: static light scattering
MBP: maltose-binding protein
ELP: elastin-like peptide
TEV: tobacco etch virus
Ni-NTA: nickel-nitrilotriacetic acid
PML: promyelocytic leukemia.
