# Supplementary material for: Intrinsically disordered sequences enable modulation of protein phase separation through distributed tyrosine motifs
Source: J Biol Chem. 2017 Sep 18;292(46):19110–20. doi: 10.1074/jbc.M117.800466 (PMC5704491; doi:10.1074/jbc.M117.800466)
Supplement: Supplemental Data [file supp_292_46_19110__index.html]

Intrinsically disordered sequences enable modulation of protein phase separation through distributed tyrosine motifs — Intrinsically disordered sequences enable modulation of protein phase separation through distributed tyrosine motifs — Disordered regions alter protein phase separation — Supplemental Data 

# Intrinsically disordered sequences enable modulation of protein phase separation through distributed tyrosine motifs

## Supplemental Data

- Full Supplemental Materials (.pdf, 4.4 MB) - Supplemental Figures S1-S8 and Table S1
