# Supplementary material for: Intrinsically disordered sequences enable modulation of protein phase separation through distributed tyrosine motifs
Source: J Biol Chem. 2017 Sep 18;292(46):19110–20. doi: 10.1074/jbc.M117.800466 (PMC5704491; doi:10.1074/jbc.M117.800466)
Supplement: Supplemental Data [file 10.1074_M117.800466_jbc.M117.800466-1.pdf]

Supplemental Materials

Intrinsically Disordered Sequences Enable Modulation of Protein Phase Separation Through Distributed Tyrosine Motifs

Yuan Lin, Simon L. Currie, Michael K. Rosen

Figure	Page
Figure S1. Droplets of $SH3_3$-FUS(27F) + PRM_4	S-2
Figure S2. FUS(27L) does not interfere with the interaction between SH3 and PRM	S-3
Figure S3. Concentration-dependent diffusion coefficients of $SH3_3$-FUS	S-4
Figure S4. Concentration-dependent diffusion coefficients of PTB and PTB-FUS(27S)	S-5
Figure S5. Scattering second virial coefficients of $SH3_3$-FUS and PTB-FUS proteins	S-6
Figure S6. $SH3_3$-FUS(WT) but not $SH3_3$ can be phosphorylated by DNA-PK	S-7
Figure S7. LLPS of $SH3_3$-FUS(WT) + PRM_4 has an upper critical solution temperature	S-8
Figure S8. Determination of the droplet concentration of $SH3_3$-FUS(WT)	S-9
Table S1. Sequences of proteins and RNA used in this study	S-10

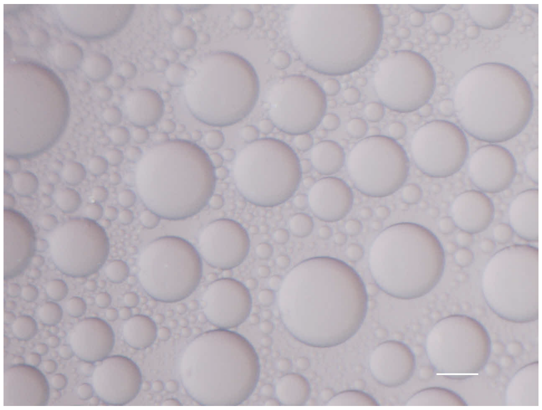

Figure S1. Liquid droplets were observed by bright-field microscopy with 133 µM of $SH3_3$-FUS(27F) and 100 µM PRM_4 (molecule concentration). Scale bar, 100 µm.

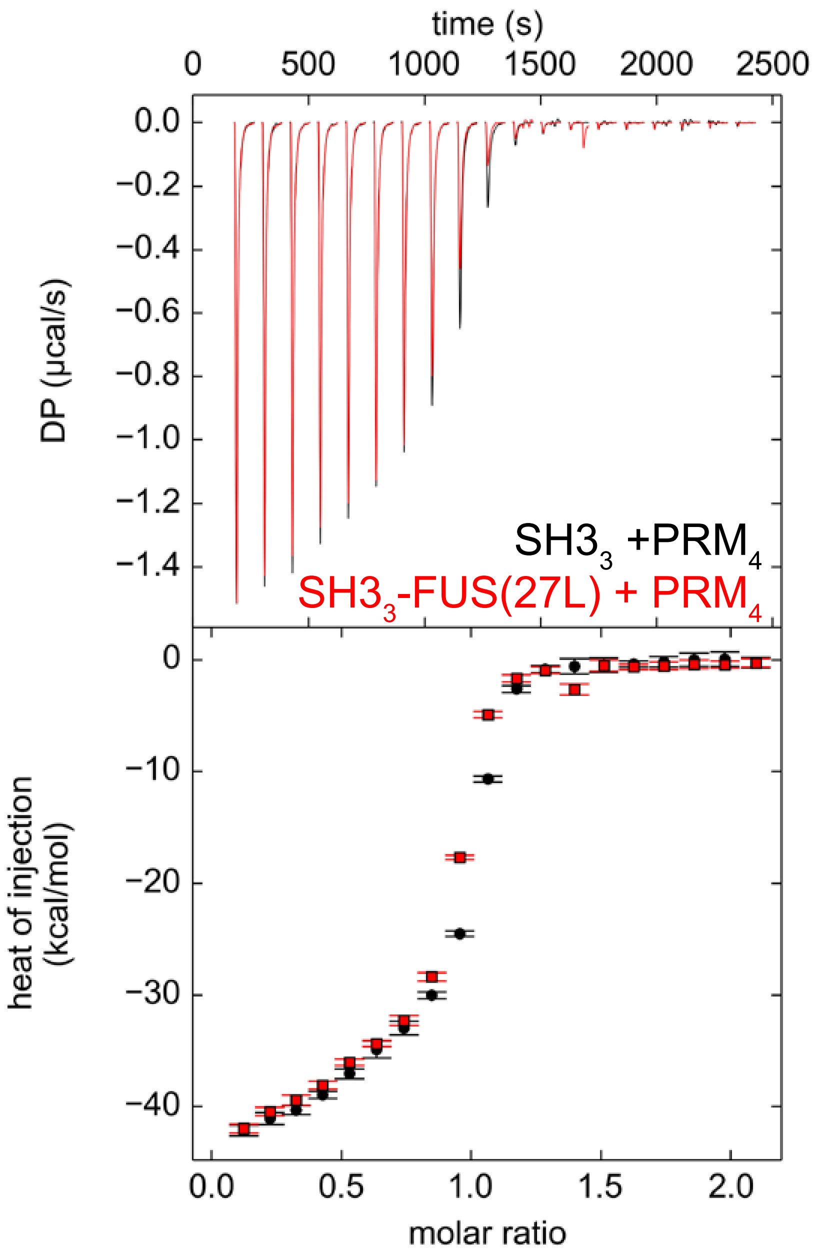

Figure S2. FUS(27L) does not interfere with the interaction between SH3 and PRM. Isothermal titration calorimetry analysis of the binding of SH3$_3$ or SH3$_3$-FUS(27L) to PRM$_4$. ~200 µM PRM$_4$ was titrated into 20 µM SH3$_3$ (*black*) or SH3$_3$-FUS(27L) (*red*) (molecule concentration). At such concentrations, phase separation did not occur. Thermogram (*upper*) and isotherm (*lower*) are shown. DP, differential power.

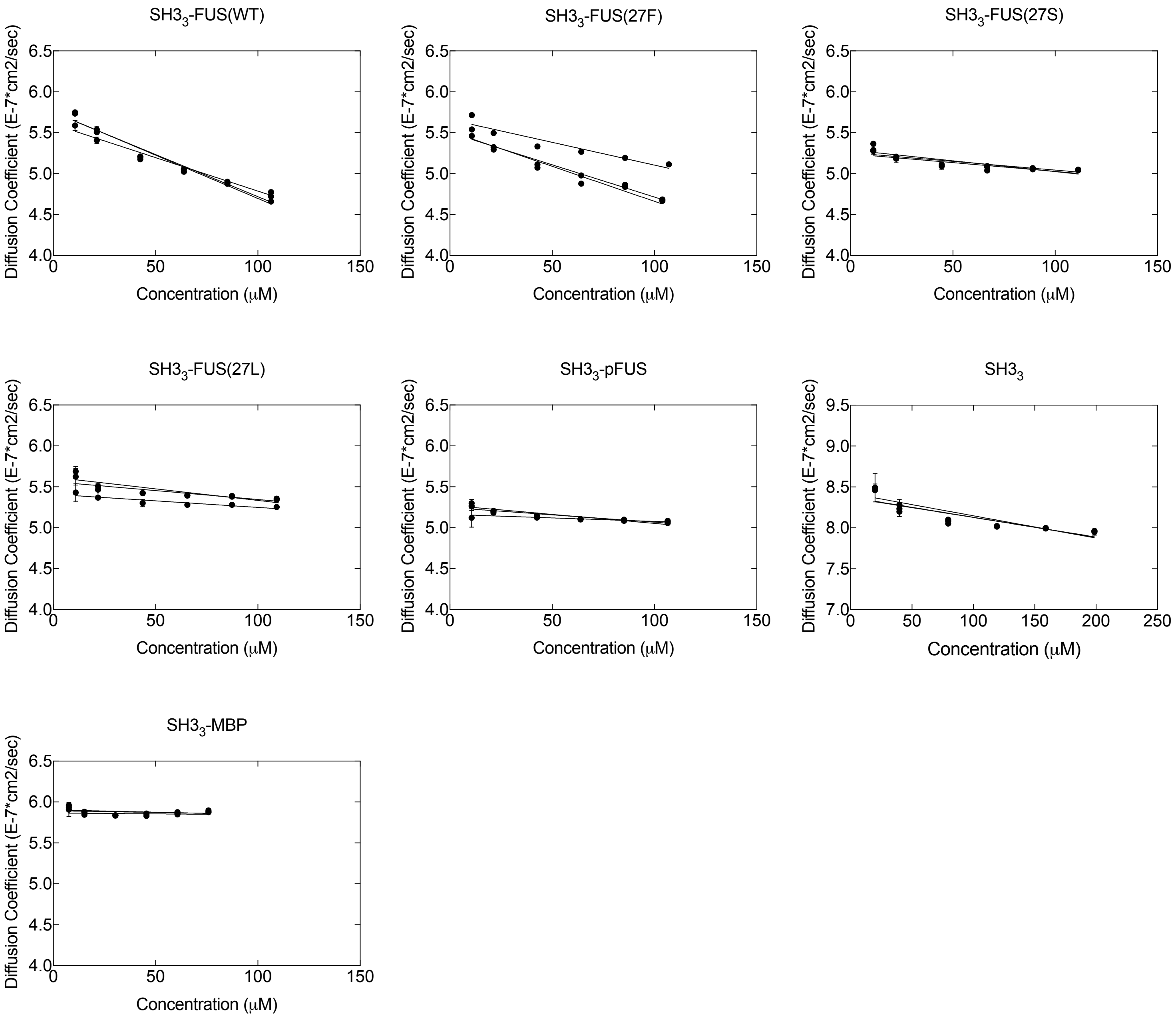

Figure S3. Concentration-dependent diffusion coefficients of SH3$_3$-FUS. Diffusion coefficients of various SH3$_3$-FUS proteins at different molecule concentrations were measured by DLS (each data point represents means of 3 technical replicates, ± s.d.). The experiments were repeated with 3 independent replicates in total. Individual linear regression was performed for each independent replicate.

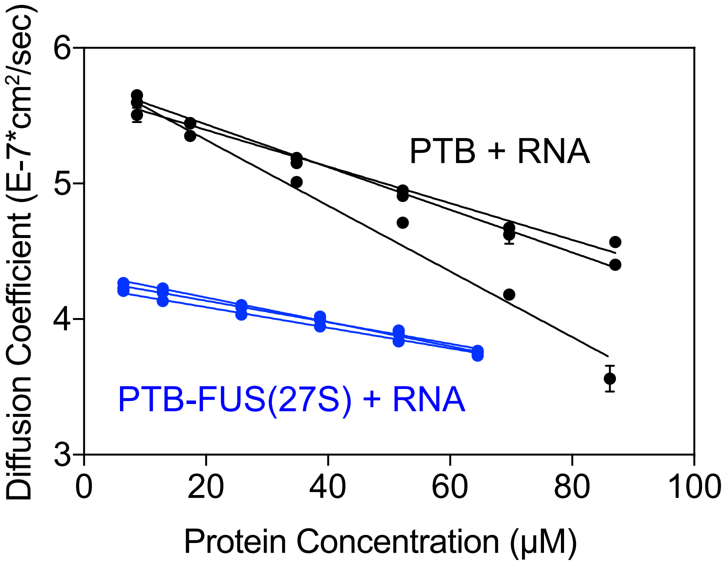

Figure S4. Concentration-dependent diffusion coefficients of PTB and PTB-FUS(27S). Diffusion coefficients of PTB (*black*) and PTB-FUS(27S) (*blue*) at different molecule concentrations were measured by DLS (each data point represents means of 3 technical replicates, ± s.d.). The experiments were repeated with 3 independent replicates in total. Individual linear regression was performed for each independent replicate.

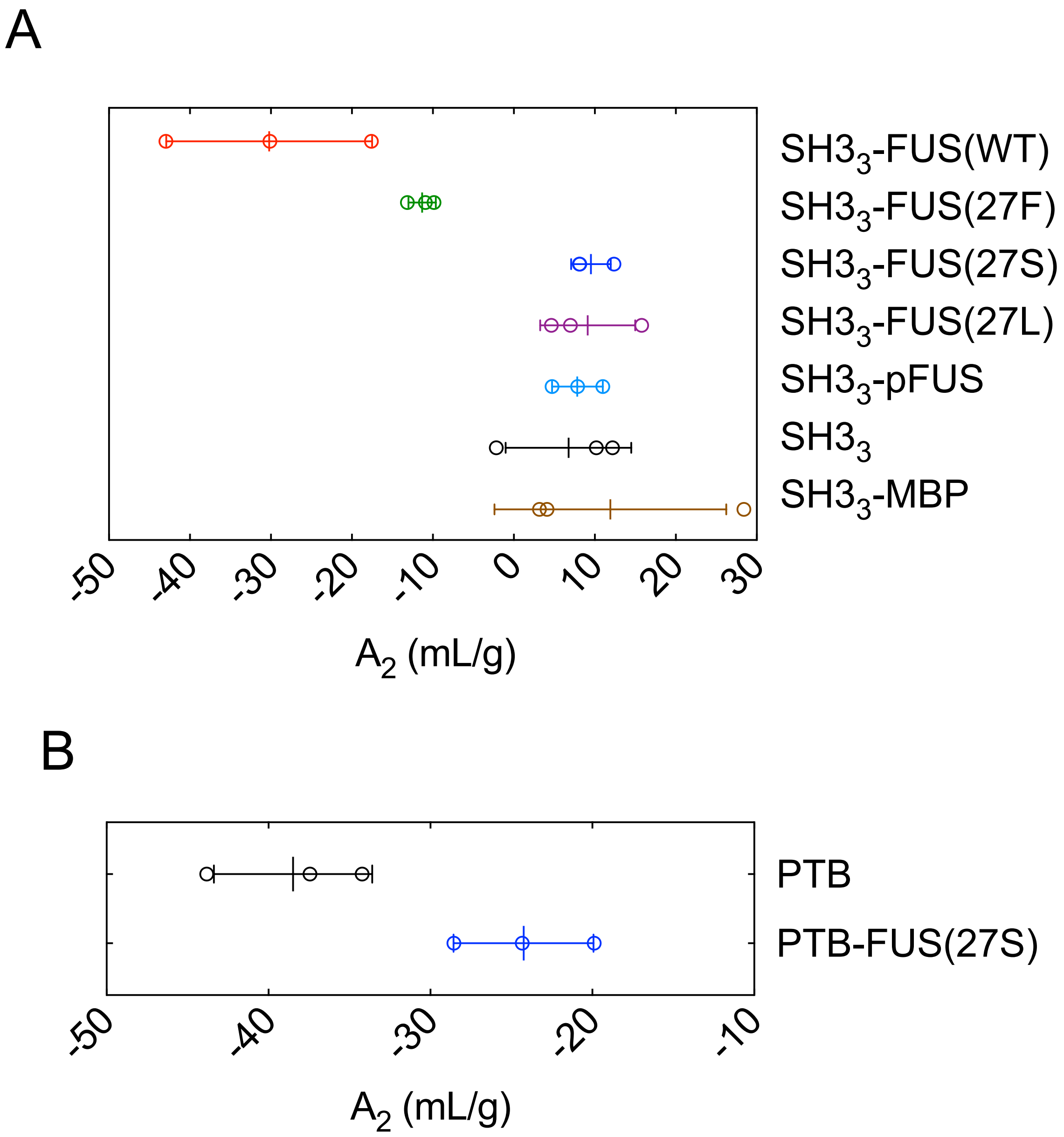

Figure S5. Scattering second virial coefficients of SH3$_3$-FUS and PTB-FUS proteins. (A) Scattering second virial coefficients of various SH3$_3$-FUS proteins based on molecule molar concentration, determined by static light scattering (means of 3 independent replicates, ± s.d.). (B) Scattering second virial coefficients of PTB and PTB-FUS(27S) based on molecule molar concentration, determined by static light scattering (means of 3 independent replicates, ± s.d.).

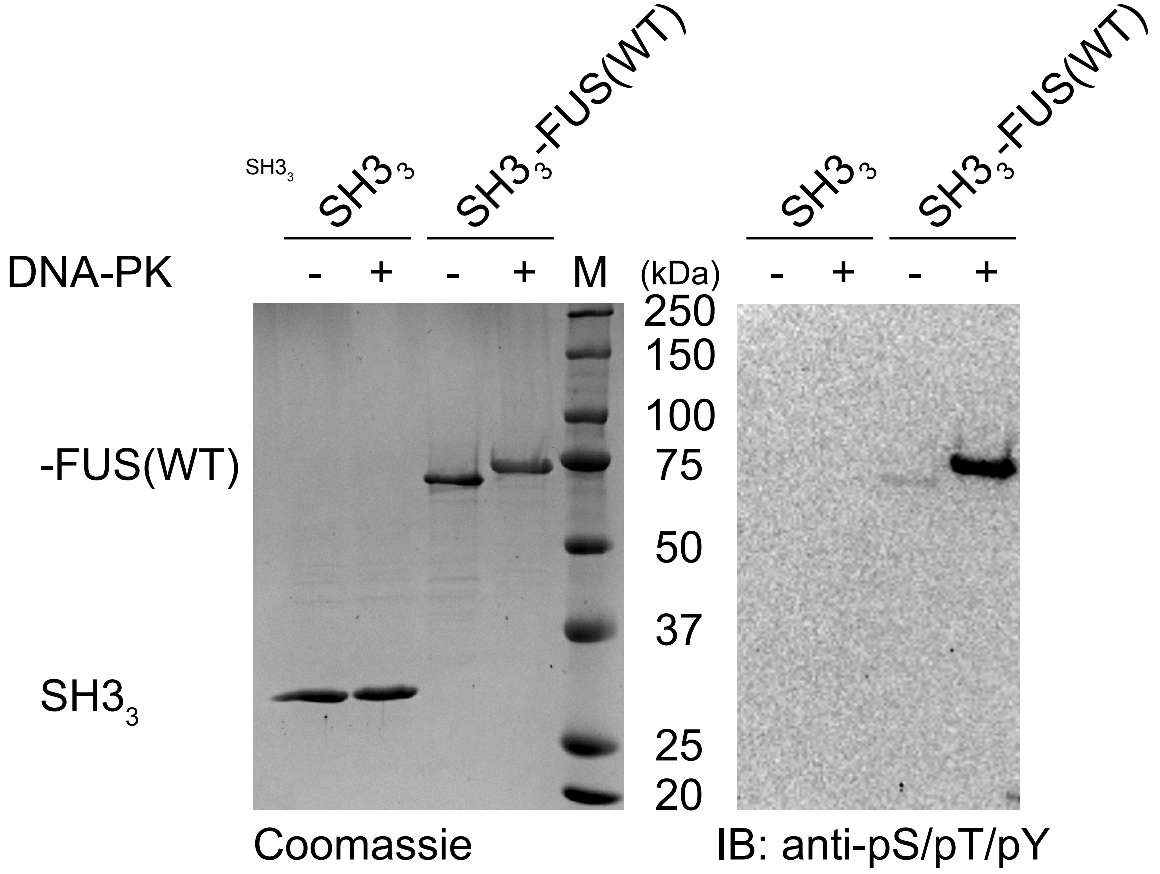

Figure S6. $SH3_3$-FUS(WT) but not $SH3_3$ can be phosphorylated by DNA-PK. Phosphorylation of $SH3_3$ and $SH3_3$-FUS(WT) by DNA-PK were evaluated by band shift on SDS-PAGE gel and western blot with an anti-phosphoserine/phosphothreonine/phosphotyrosine antibody, compared to untreated samples. $SH3_3$-FUS(WT) treated with DNA-PK appeared as a higher band than the untreated protein, which can be recognized by anti-phosphoserine/phosphothreonine/phosphotyrosine antibody. However, there was no difference in band position between DNA-PK treated and untreated $SH3_3$, and neither can be recognized by antibody.

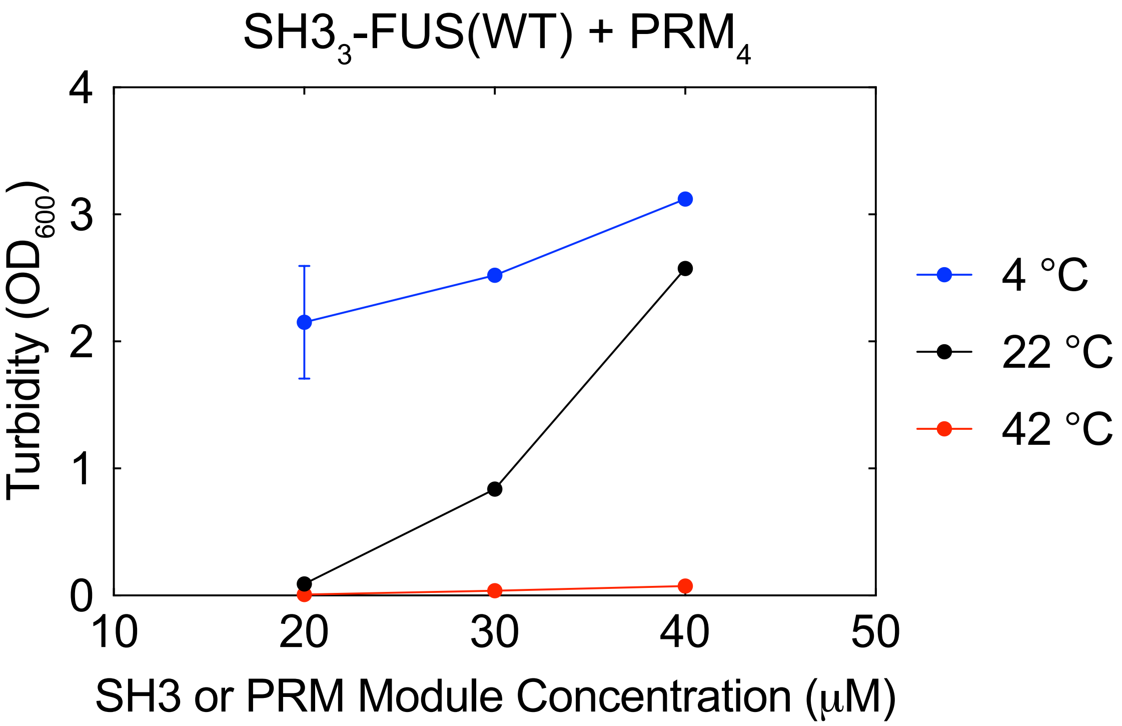

Figure S7. LLPS of SH3$_3$-FUS(WT) plus PRM$_4$ is favored at lower temperature but disfavored at higher temperature, indicating the system has upper critical solution temperature (UCST). The effect of temperature on LLPS of SH3$_3$-FUS(WT) + PRM$_4$, evaluated by turbidity. OD$_{600}$ at different module concentrations and temperatures is plotted as mean ± s.d., from three independent measurements. The module concentration of SH3 was equal to that of PRM in each sample. 4 °C, *blue*; 22 °C, *black*; 42 °C, *red*.

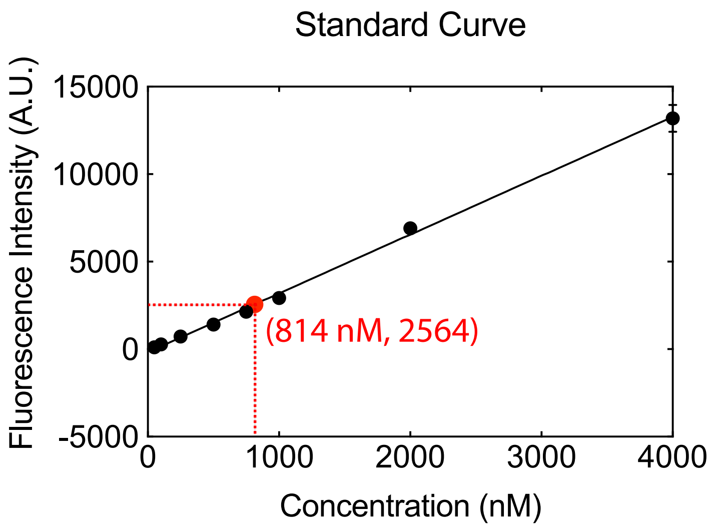

	Concentration
Labeled $SH3_3$-FUS(WT) $_{total}$	0.05 μM
Unlabeld $SH3_3$-FUS(WT) $_{total}$	20 μM
Labeled $SH3_3$-FUS(WT) $_{droplet}$	0.814 μM
Unlabeld $SH3_3$-FUS(WT) $_{droplet}$	326 μM

Figure S8. Determination of the droplet concentration of $SH3_3$-FUS(WT). 20 μM $SH3_3$-FUS(WT), 50 nM $SH3_3$-FUS(WT) labeled with Oregon Green 488, and 15 μ M PRM_4 (molecule concentration) were mixed to induce LLPS. The droplet concentration of labeled $SH3_3$-FUS(WT) (*red dot, 814 nM*) was calculated from the average droplet intensities (*red dot, 2564*) and a standard curve of a series of pure Oregon Green 488 at different concentrations (*black dots*). Since the ratio between total labeled and total unlabeled $SH3_3$-FUS(WT) was known, the droplet concentration of unlabeled $SH3_3$-FUS(WT) can be calculated from that of labeled $SH3_3$-FUS(WT). The total and droplet concentrations of labeled and unlabeled proteins are summarized in the table on the right.

Table S1. Sequences of proteins and RNA used in this study.

Protein Name	Sequence	Note
$SH3_3$	GCHMDLNMPAYVKFNYMAEREDELSLIKGTKVIV MEKSSDGWWRGSYNGQVGWFPSNYVTEEGDSPLA SGAGGSEGGGSEGGTSGATDLNMPAYVKFNYMAE REDELSLIKGTKVIVMEKSSDGWWRGSYNGQVGW FPSNYVTEEGDSPLASGAGGSEGGGSEGGTSGATDL NMPAYVKFNYMAEREDELSLIKGTKVIVMEKSSDG WWRGSYNGQVGWFPSNYVTEEGDSPL	SH3 derived from the human Nck1 second SH3 domain (residues 106-168, C139S)
$SH3_3$-FUS(WT)	GCHMDLNMPAYVKFNYMAEREDELSLIKGTKVIV MEKSSDGWWRGSYNGQVGWFPSNYVTEEGDSPLA SGAGGSEGGGSEGGTSGATDLNMPAYVKFNYMAE REDELSLIKGTKVIVMEKSSDGWWRGSYNGQVGW FPSNYVTEEGDSPLASGAGGSEGGGSEGGTSGATDL NMPAYVKFNYMAEREDELSLIKGTKVIVMEKSSDG WWRGSYNGQVGWFPSNYVTEEGDSPLGGSGGSAS NDYTQQATQSYGAYPTQPGQGYSQQSSQPYGQQS YSGYSQSTDTSGYGQSSYSSYGQSQNTGYGTQSTP QGYGSTGGYGSSQSSQSSYGQQSSYPGYGQQPAPSS TSGSYGSSSQSSSYGQPQSGSYSQQPSYGGQQQSYG QQQSYNPPQGYGQQNQYNSSSGGGGGGGGGNYG QDQSSMSSGGGSGGGYGNQDQSGGGGSGGYGQQD RG	FUS_{IDR} derived from human FUS(residues 1-214)
$SH3_3$-FUS(27S)	GCHMDLNMPAYVKFNYMAEREDELSLIKGTKVIV MEKSSDGWWRGSYNGQVGWFPSNYVTEEGDSPLA SGAGGSEGGGSEGGTSGATDLNMPAYVKFNYMAE REDELSLIKGTKVIVMEKSSDGWWRGSYNGQVGW FPSNYVTEEGDSPLASGAGGSEGGGSEGGTSGATDL NMPAYVKFNYMAEREDELSLIKGTKVIVMEKSSDG WWRGSYNGQVGWFPSNYVTEEGDSPLGGSGGSAS NDSTQQATQSSGASPTQPGQGSSQQSSQPSGQQSSS GSSQSTDTSGSGQSSSSSSGQSQNTGSGTQSTPQGSG STGGSGSSQSSQSSSGQQSSSPGSGQQPAPSSTSGSS GSSSQSSSSGQPQSGSSSQQPSSGGQQQSSGQQQSS NPPQGSGQQNQSNSSSGGGGGGGGGNSGQDQSS MSSGGGSGGGSGNQDQSGGGGSGGSGQQDRG	Tyrosine residues in FUS_{IDR} are all mutated to serine residues
$SH3_3$-FUS(27L)	GCHMDLNMPAYVKFNYMAEREDELSLIKGTKVIV MEKSSDGWWRGSYNGQVGWFPSNYVTEEGDSPLA SGAGGSEGGGSEGGTSGATDLNMPAYVKFNYMAE REDELSLIKGTKVIVMEKSSDGWWRGSYNGQVGW FPSNYVTEEGDSPLASGAGGSEGGGSEGGTSGATDL NMPAYVKFNYMAEREDELSLIKGTKVIVMEKSSDG WWRGSYNGQVGWFPSNYVTEEGDSPLGGSGGSMA SNDLTQQATQSLGALPTQPGQGLSQQSSQPLGQQSL	Tyrosine residues in FUS_{IDR} are all mutated to leucine residues

	SGLSQSTDTSGLGQSSLSSLGQSQNTGLGTQSTPQG<br>LGSTGGLGSSQSSQSSLGQQSSLPGLGQQPAPSSTSG<br>SLGSSSQSSSLGQPQSGSLSQQPSLGGQQQSLGQQQ<br>SLNPPQGLGQQNQLNSSSGGGGGGGGGNLGQDQ<br>SSMSSGGGSGGGLGNQDQSGGGGSGGLGQQDRG	
SH3$_3$-FUS(27F)	GCHMDLNMPAYVKFNYMAEREDELSLIKGTKVIV<br>MEKSSDGWWRGSYNGQVGWFPSNYVTEEGDSPLA<br>SGAGGSEGGGSEGGTSGATDLNMPAYVKFNYMAE<br>REDELSLIKGTKVIVMEKSSDGWWRGSYNGQVGW<br>FPSNYVTEEGDSPLASGAGGSEGGGSEGGTSGATDL<br>NMPAYVKFNYMAEREDELSLIKGTKVIVMEKSSDG<br>WWRGSYNGQVGWFPSNYVTEEGDSPLGGSGGSMA<br>SNDFTQQATQSFGAFPTQPGQGFSQQSSQPFGQQSF<br>SGFSQSTDTSGFGQSSFSSFGQSQNTGFGTQSTPQGF<br>GSTGGFGSSQSSQSSFGQQSSFPGFGQQPAPSSTSGS<br>FGSSSQSSSFGQPQSGSFSQQPSFGGQQQSFGQQQSF<br>NPPQGFGQQNQFNSSSGGGGGGGGGNFGQDQSS<br>MSSGGGSGGGFGNQDQSGGGGSGGFGQQDRG	Tyrosine residues in FUS$_{IDR}$ are all mutated to phenylalanine residues
SH3$_3$-FUS(5S-1)	GCHMDLNMPAYVKFNYMAEREDELSLIKGTKVIV<br>MEKSSDGWWRGSYNGQVGWFPSNYVTEEGDSPLA<br>SGAGGSEGGGSEGGTSGATDLNMPAYVKFNYMAE<br>REDELSLIKGTKVIVMEKSSDGWWRGSYNGQVGW<br>FPSNYVTEEGDSPLASGAGGSEGGGSEGGTSGATDL<br>NMPAYVKFNYMAEREDELSLIKGTKVIVMEKSSDG<br>WWRGSYNGQVGWFPSNYVTEEGDSPLGGSGGSMA<br>SNDSTQQATQSSGASPTQPGQGSSQQSSQPSGQQSY<br>SGYSQSTDTSGYGQSSYSSYGQSQNTGYGTQSTPQ<br>GYGSTGGYGSSQSSQSSYGQQSSYPGYGQQPAPSST<br>SGSYGSSSQSSSYGQPQSGSYSQQPSYGGQQQSYGQ<br>QQSYNPPQGYGQQNQYNSSSGGGGGGGGGNYGQ<br>DQSSMSSGGGSGGGYGNQDQSGGGGSGGYGQQDR<br>G	
SH3$_3$-FUS(5S-2)	GCHMDLNMPAYVKFNYMAEREDELSLIKGTKVIV<br>MEKSSDGWWRGSYNGQVGWFPSNYVTEEGDSPLA<br>SGAGGSEGGGSEGGTSGATDLNMPAYVKFNYMAE<br>REDELSLIKGTKVIVMEKSSDGWWRGSYNGQVGW<br>FPSNYVTEEGDSPLASGAGGSEGGGSEGGTSGATDL<br>NMPAYVKFNYMAEREDELSLIKGTKVIVMEKSSDG<br>WWRGSYNGQVGWFPSNYVTEEGDSPLGGSGGSMA<br>SNDYTQQATQSYGAYPTQPGQGYSQQSSQPYGQQS<br>SSGSSQSTDTSGSGQSSSSSSGQSQNTGYGTQSTPQG<br>YGSTGGYGSSQSSQSSYGQQSSYPGYGQQPAPSSTS<br>GSYGSSSQSSSYGQPQSGSYSQQPSYGGQQQSYGQ<br>QQSYNPPQGYGQQNQYNSSSGGGGGGGGGNYGQ<br>DQSSMSSGGGSGGGYGNQDQSGGGGSGGYGQQDR<br>G	
SH3$_3$-FUS(5S-3)	GCHMDLNMPAYVKFNYMAEREDELSLIKGTKVIV<br>MEKSSDGWWRGSYNGQVGWFPSNYVTEEGDSPLA<br>SGAGGSEGGGSEGGTSGATDLNMPAYVKFNYMAE	

	REDELSLIKGTKVIVMEKSSDGWWRGSYNGQVGW<br>FPSNYVTEEGDSPLASGAGGSEGGGSEGGTSGATDL<br>NMPAYVKFNYMAEREDELSLIKGTKVIVMEKSSDG<br>WWRGSYNGQVGWFPSNYVTEEGDSPLGGSGGSMA<br>SNDYTQQATQSYGAYPTQPGQGYSQQSSQPYGQQS<br>YSGYSQSTDTSGYGQSSYSSYGQSQNTGSGTQSTPQ<br>GSGSTGGSGSSQSSQSSSGQQSSSPGYGQQPAPSSTS<br>GSYGSSSQSSSYGQPQSGSYSQQPSYGGQQQSYGQ<br>QQSYNPPQGYGQQNQYNSSSGGGGGGGGGGNYGQ<br>DQSSMSSGGGSGGGYGNQDQSGGGGSGGYGQQDR<br>G	
SH3$_3$-<br>FUS(5S-4)	GCHMDLNMPAYVKFNYMAEREDELSLIKGTKVIV<br>MEKSSDGWWRGSYNGQVGWFPSNYVTEEGDSPLA<br>SGAGGSEGGGSEGGTSGATDLNMPAYVKFNYMAE<br>REDELSLIKGTKVIVMEKSSDGWWRGSYNGQVGW<br>FPSNYVTEEGDSPLASGAGGSEGGGSEGGTSGATDL<br>NMPAYVKFNYMAEREDELSLIKGTKVIVMEKSSDG<br>WWRGSYNGQVGWFPSNYVTEEGDSPLGGSGGSMA<br>SNDYTQQATQSYGAYPTQPGQGYSQQSSQPYGQQS<br>YSGYSQSTDTSGYGQSSYSSYGQSQNTGYGTQSTP<br>QGYGSTGGYGSSQSSQSSYGQQSSYPGSGQQPAPSS<br>TSGSSGSSSQSSSSGQPQSGSSSQQPSSGGQQQSYGQ<br>QQSYNPPQGYGQQNQYNSSSGGGGGGGGGGNYGQ<br>DQSSMSSGGGSGGGYGNQDQSGGGGSGGYGQQDR<br>G	
SH3$_3$-<br>FUS(5S-5)	GCHMDLNMPAYVKFNYMAEREDELSLIKGTKVIV<br>MEKSSDGWWRGSYNGQVGWFPSNYVTEEGDSPLA<br>SGAGGSEGGGSEGGTSGATDLNMPAYVKFNYMAE<br>REDELSLIKGTKVIVMEKSSDGWWRGSYNGQVGW<br>FPSNYVTEEGDSPLASGAGGSEGGGSEGGTSGATDL<br>NMPAYVKFNYMAEREDELSLIKGTKVIVMEKSSDG<br>WWRGSYNGQVGWFPSNYVTEEGDSPLGGSGGSMA<br>SNDYTQQATQSYGAYPTQPGQGYSQQSSQPYGQQS<br>YSGYSQSTDTSGYGQSSYSSYGQSQNTGYGTQSTP<br>QGYGSTGGYGSSQSSQSSYGQQSSYPGYGQQPAPSS<br>TSGSYGSSSQSSSYGQPQSGSYSQQPSYGGQQQSSG<br>QQQSSNPPQGSGQQNQSNSSSGGGGGGGGGGNSG<br>QDQSSMSSGGGSGGGYGNQDQSGGGGSGGYGQQD<br>RG	
SH3$_3$-<br>FUS(5S-6)	GCHMDLNMPAYVKFNYMAEREDELSLIKGTKVIV<br>MEKSSDGWWRGSYNGQVGWFPSNYVTEEGDSPLA<br>SGAGGSEGGGSEGGTSGATDLNMPAYVKFNYMAE<br>REDELSLIKGTKVIVMEKSSDGWWRGSYNGQVGW<br>FPSNYVTEEGDSPLASGAGGSEGGGSEGGTSGATDL<br>NMPAYVKFNYMAEREDELSLIKGTKVIVMEKSSDG<br>WWRGSYNGQVGWFPSNYVTEEGDSPLGGSGGSMA<br>SNDYTQQATQSYGAYPTQPGQGYSQQSSQPYGQQS<br>YSGYSQSTDTSGYGQSSYSSYGQSQNTGYGTQSTP<br>QGYGSTGGYGSSQSSQSSYGQQSSYPGYGQQPAPSS<br>TSGSYGSSSQSSSYGQPQSGSYSQQPSYGGQQQSYG<br>QQQSYNPPQGSGQQNQSNSSSGGGGGGGGGGNSG	

	QDQSSMSSGGGSGGGSGNQDQSGGGGSGGSGQQD RG	
$SH3_3$-MBP	GCHMDLNMPAYVKFNYMAEREDELSLIKGTKVIV MEKSSDGWWRGSYNGQVGWFPSNYVTEEGDSPLA SGAGGSEGGGSEGGTSGATDLNMPAYVKFNYMAE REDELSLIKGTKVIVMEKSSDGWWRGSYNGQVGW FPSNYVTEEGDSPLASGAGGSEGGGSEGGTSGATDL NMPAYVKFNYMAEREDELSLIKGTKVIVMEKSSDG WWRGSYNGQVGWFPSNYVTEEGDSPLGGSGGSMK IEEGKLVIWINGDKGYNGLAEVGKKFEKDTGIKVT VEHPDKLEEKFPQVAATGDGPDIIFWAHDRFGGYA QSGLLAEITPDKAFQDKLYPFTWDAVRYNGKLIAY PIAVEALSLIYNKDLLPNPPKTWEEIPALDKELKAKG KSALMFNLQEPYFTWPLIAADGGYAFKYENGKYDI KDVGVDNAGAKAGLTFLVDLIKNKHMNADTDYSI AEAAFNKGETAMTINGPWAWSNIDTSKVNYGVTV LPTFKGQPSKPFVGVLSAGINAASPNKELAKEFLEN YLLTDEGLEAVNKDKPLGAVALKSYEEELAKDPRI AATMENAQKGEIMPNIPQMSAFWYAVRTAVINAAS GRQTVDEALKDAQT	
FUS(WT)	MASNDYTQQATQSYGAYPTQPGQGYSQQSSQPYG QQSYSGYSQSTDTSGYGQSSYSSYGQSQNTGYGTQ STPQGYGSTGGYGSSQSSQSSYGQQSSYPGYGQQP APSSTSGSYGSSSQSSSYGQPQSGSYSQQPSYGGQQ QSYGQQQSYNPPQGYGQQNQYNSSSGGGGGGGG GNYGQDQSSMSSGGGSGGGYGNQDQSGGGGSGGY GQQDRG	
FUS(27S)	MASNDSTQQATQSSGASPTQPGQGSSQQSSQPSGQ QSSSGSSQSTDTSGSGQSSSSSSGQSQNTGSGTQSTP QGSGSTGGSGSSQSSQSSSGQQSSSPGSGQQPAPSST SGSSGSSSQSSSSGQPQSGSSSQQPSSGGQQQSSGQQ QSSNPPQGSGQQNQSNSSSGGGGGGGGGGNSGQD QSSMSSGGGSGGGSGNQDQSGGGGSGGSGQQDRG	
PRM_4	GHMCGGSWGGSKKKKTAPTPPKRSGGSGGSGGSG GSKKKKTAPTPPKRSGGSGGSGGSGGSKKKKTAPT PPKRSGGSGGSGGSGGSKKKKTAPTPPKRSGGSGSE NLYFQ	PRM derived from ABL1 (residues 606-618)
PTB	GHMDGIVPDIAVGTKRGSDELFSTCVTNGPFIMSSN SASAANGNDSKKFKGDSRSAGVPSRVIHIRKLPIDV TEGEVISLGLPFGKVTNLLMLKGKNQAFIEMNTEEA ANTMVNYYTSVTPVLRGQPIYIQFSNHKELKTDSSP NQARAQAALQAVNSVQSGNLALAASAAAVDAGM AMAGQSPVLRIIVENLFYPVTLDVLHQIFSKFGTVL KIITFTKNNQFQALLQYADPVSAQHAKLSLDGQNIY NACCTLRIDFSKLTSLNVKYNNDKSRDYTRPDLPSG DSQPSLDQTMAAAFGLSVPNVHGALAPLAIPSAAA AAAAAGRIAIPGLAGAGNSVLLVSNLNPERVTPQSL FILFGVYGDVQRVKILFNKKENALVQMADGNQAQL AMSHLNGHKLHGKPIRITLSKHQNVQLPREGQEDQ	PTB derived from human PTB(residu es 1-531)

	GLTKDYGNSPLHRFKKPGSKNFQNIFPPSATLHLSNI<br>PPSVSEEDLKVLFSSNGGVVKGFKFFQKDRKMALIQ<br>MGSVEEAVQALIDLHNHDLGENHHLRVSFSKSTI	
PTB-<br>FUS(27S)	GHMDGIVPDIAVGTKRGSDELFSTCVTNGPFIMSSN<br>SASAANGNDSKKFKGDSRSAGVPSRVIHIRKLPIDV<br>TEGEVISLGLPFGKVTNLLMLKGKNQAFIEMNTEEA<br>ANTMVNYYTSVTPVLRGQPIYIQFSNHKELKTDSSP<br>NQARAQAALQAVNSVQSGNLALAASAAAVDAGM<br>AMAGQSPVLRIIVENLFYPVTLDVLHQIFSKFGTVL<br>KIITFTKNNQFQALLQYADPVSAQHAKLSLDGQNIY<br>NACCTLRIDFSKLTSLNVKYNNDKSRDYTRPDLPSG<br>DSQPSLDQTMAAAFGLSVPNVHGALAPLAIPSAAA<br>AAAAAGRIAIPGLAGAGNSVLLVSNLNPERVTPQSL<br>FILFGVYGDVQRVKILFNKKENALVQMADGNQAQL<br>AMSHLNGHKLHGKPIRITLSKHQNVQLPREGQEDQ<br>GLTKDYGNSPLHRFKKPGSKNFQNIFPPSATLHLSNI<br>PPSVSEEDLKVLFSSNGGVVKGFKFFQKDRKMALIQ<br>MGSVEEAVQALIDLHNHDLGENHHLRVSFSKSTIG<br>GSAAAGGSMASNDSTQQATQSSGASPTQPGQGSSQ<br>QSSQPSGQQSSSGSSQSTDTSGSGQSSSSSSGQSQNT<br>GSGTQSTPQGSGSTGGSGSSQSSQSSSGQQSSSPGSG<br>QQPAPSSTSGSSGSSSQSSSSGQPQSGSSSQQPSSGG<br>QQQSSGQQQSSNPPQGSGQQNQSNSSSGGGGGGGG<br>GGNSGQDQSSMSSGGGSGGGSGNQDQSGGGGSGG<br>SGQQDRG	
RNA	$(UCUCUAAAAA)_5$	PTB<br>pentameric<br>ligand
